# The neural mechanisms for the recognition of face identity in humans

**DOI:** 10.3389/fpsyg.2014.00672

**Published:** 2014-06-26

**Authors:** Stefano Anzellotti, Alfonso Caramazza

**Affiliations:** ^1^Department of Psychology, Harvard UniversityCambridge, MA, USA; ^2^Center for Mind/Brain Sciences, University of TrentoTrento, Italy

**Keywords:** faces, identity, fMRI, object recognition, invariance

## Abstract

Every day we encounter dozens of people, and in order to interact with them appropriately we need to recognize their identity. The face is a crucial source of information to recognize a person’s identity. However, recognizing the identity of a face is challenging because it requires distinguishing between very similar images (e.g., the front views of two different faces) while categorizing very different images (e.g., a front view and a profile) as the same person. Neuroimaging has the whole-brain coverage needed to investigate where representations of face identity are encoded, but it is limited in terms of spatial and temporal resolution. In this article, we review recent neuroimaging research that attempted to investigate the representation of face identity, the challenges it faces, and the proposed solutions, to conclude that given the current state of the evidence the right anterior temporal lobe is the most promising candidate region for the representation of face identity.

## INTRODUCTION

In this paper, we focus on recent neuroimaging research that has investigated aspects of the neural mechanisms underlying the perceptual recognition of face identity. The ability to recognize individuals is crucial for guiding behavior – it allows us to retrieve information about people and interact with them in appropriate ways. Many different cues can be used to recognize an individual, including the appearance of the face, the sound of the voice, as well as the context in which we encounter a person and prior knowledge about his/her current general location (see Oliva and Torralba, 2007; Goesaert and Op de Beeck, 2013). A promising approach consists in studying how each of these cues is processed when other cues are controlled, to then proceed with an investigation of how the different cues are integrated. Among the different cues that can be used for person recognition, the face is a crucial source of information and is usually sufficient in isolation to recognize a person’s identity. However, recognizing face identity is also computationally challenging: it requires discounting identity-irrelevant changes in sensory stimulation (such as changes in viewpoint and illumination) without losing the ability to perform fine-grained discriminations needed to distinguish the faces of similar individuals.

The earliest insights into the neural mechanisms underlying the ability to recognize face identity came from the study of patients with selective impairment for the recognition of faces (Charcot, 1883; Wilbrand, 1892; Heidenhain, 1927; Jossmann, 1929), which was subsequently named prosopagnosia (Bodamer, 1947). Hecaen and Angelergues (1962) investigated the location of lesions producing selective deficits for faces in a group of 22 patients, and observed that prosopagnosic patients tended to have lesions in the right hemisphere, often involving occipital regions. A review of the neuropsychological literature individuated the right occipitotemporal cortex as the most common location of the lesion in prosopagnosic patients (Meadows, 1974). Convergent evidence in support of the view that damage to the occipitotemporal cortex leads to prosopagnosia was reported in several studies (Whiteley and Warrington, 1977; Damasio et al., 1982; Malone et al., 1982).

Other neuropsychological studies reported deficits for the recognition of familiar and famous faces in patients with herpes simplex encephalitis (Warrington and Shallice, 1984; Warrington and McCarthy, 1988) and semantic dementia (Snowden et al., 2004), with more frequent face recognition deficits in the right than in the left temporal variant of semantic dementia (Thompson et al., 2003). These pathologies affect the anterior portions of the temporal lobe (Kapur et al., 1994; Mummery et al., 2000; Gitelman et al., 2001; Hodges and Patterson, 2007; Noppeney et al., 2007). Furthermore, the highest lesion overlap in patients with face recognition deficits was found the be in the right anterior temporal lobe (Tranel et al., 1997). Consistent with the neuropsychological literature, neuroimaging studies in healthy participants individuated regions showing stronger activity for faces than for other kinds of objects in occipitotemporal cortex [occipital face area (OFA) and fusiform face area (FFA); Sergent et al., 1992; Puce et al., 1996; Kanwisher et al., 1997; Gauthier et al., 2000; see Çukuret al., 2013 for an in-depth analysis of voxel response profiles] and the anterior temporal lobes (Rajimehr et al., 2009).

Both occipitotemporal regions and anterior temporal regions show stronger activity for faces than other objects, and lesions in these regions lead to face processing deficits. What are the respective contributions of the two brain regions in representing face identity? The finding that lesion to a brain region leads to a deficit for face recognition does not imply that that region encodes representations of face identity – it might just provide necessary input to another region that represents face identity. At the same time, neither occipitotemporal nor anterior temporal regions seem to be involved merely in the processing of “low level” perceptual details. Patients with anterior temporal lesions have intact basic perceptual abilities (Warrington and Shallice, 1984), and while patients with occipitotemporal lesions often have visual field defects (Meadows, 1974), they are able to describe and draw individual face parts (Bodamer, 1947). A deeper understanding of the properties of representations in these regions is needed to clarify their respective roles for the recognition of face identity. This paper is concerned with the neuroimaging research pursuing this understanding. In particular, the focus is on perceptual representations of face identity, rather than on other aspects of person identity such as associated semantic knowledge (Tsukiura et al., 2002), or the sense of familiarity and emotional responses which can be impaired in disorders such as Capgras syndrome (Ellis and Lewis, 2001).

## DISCRIMINATION OF FACE TOKENS

Before delving into the discussion of the literature, it is necessary to introduce some terms and clarify their use. We will use the term “face token” to refer to a specific image of a face, seen from a particular viewpoint and under a particular illumination. The recognition of face identity requires (1) to distinguish between face tokens that depict different people, and (2) to recognize when two different face tokens depict the same person. We will use the term “invariant face representations” to refer to representations that encode information about whether two face tokens depict the same person, for *some or all* pairs of face tokens that depict a same person. Note that invariance can be partial, for example, there might be representations that are invariant to changes in viewpoint of up to 35°. Therefore, not all invariant face representations are representations of face identity. We will reserve the term “representation of face identity” for representations that encode information that allows determining that two face tokens depict the same person for all pairs of face tokens that are recognized as a same person by a human observer. Whether or not there exists one brain region that encodes representations with invariance across all transformations that humans can generalize across is an empirical question. To search for representations of face identity, we can first search for representations that distinguish between face tokens that depict different people, and then test whether and to which extent they are invariant. Finding brain regions that distinguish between face tokens that depict different people provides us with a series of potential candidates for the representation of face identity.

The investigation of regions that distinguish between face tokens that depict different people with functional magnetic resonance imaging (fMRI) is challenging, because when properties like viewpoint and illumination are controlled, face tokens that depict different people do not produce significantly different blood-oxygen-level dependent (BOLD) responses when analyzed with standard univariate approaches. Nonetheless, fMRI remains one of the best methods available to localize regions that distinguish between face tokens that depict different people. This is because it allows coverage of a large extent of the human brain in a single study, and because among the methods with this property it is the one that offers the highest spatial resolution.

For this reason, in the course of the past two decades, researchers used fMRI to investigate the neural mechanisms underlying the recognition of face identity, developing and employing experimental designs and data analysis approaches to meet the challenge posed by the subtle differences in the BOLD responses produced by different face tokens.

One approach to individuating representations that distinguish between face tokens that depict different people involves using fMRI-adaptation (fMR-A). FMR-A is a phenomenon characterized by reduced BOLD responses to repeated stimuli (Grill-Spector et al., 1999). FMR-A has also been observed during the presentation of two stimuli that are not identical but are similar along some dimension (Grill-Spector et al., 1999; Vuilleumier et al., 2002). For example, fMR-A can occur for the presentation of different stimuli from the same category (Fairhall et al., 2011). FMR-A has been used to investigate representations of face tokens in a series of studies (Grill-Spector et al., 1999; Gauthier et al., 2000; Rotshtein et al., 2004; Furl et al., 2007). Greater adaptation for repetitions of a same face token than for the presentation of different face tokens has been observed in the FFA (Gauthier et al., 2000), as well as in occipitotemporal regions defined with a broader contrast between faces and textures (Grill-Spector et al., 1999).

As an alternative to fMR-A, some researchers have used multivariate pattern analysis (MVPA) to improve the sensitivity of fMRI (Haxby et al., 2001; Haynes and Rees, 2006). Multivariate approaches extract information from the pattern of activity in multiple voxels. They are more sensitive than univariate approaches, because they can distinguish between BOLD responses within a region that have the same mean but different spatial distributions.

A common method consists in using univariate analyses in order to individuate regions showing stronger responses to faces than other objects (“face-selective” regions) and subsequently investigate information content with MVPA within these regions. With this regions-of-interest (ROI) approach it has been shown that face-selective regions, including notably the FFA, encode information about face tokens (Nestor et al., 2011; Anzellotti et al., 2013; Goesaert and Op de Beeck, 2013; Verosky et al., 2013; but see Natu et al., 2010). However, this approach is based on the implicit assumption that localizing the brain regions showing the greatest mean difference between the activity in response to faces and the activity in response to other objects exhaustively captures the regions involved in the recognition of face identity. This assumption might not hold: there may be regions that do not show face-selectivity but still contribute to the recognition of face identity.

An alternative to the use of face selectivity is searchlight analysis (Kriegeskorte et al., 2006; Kriegeskorte and Bandettini, 2007) to individuate regions that distinguish between face tokens in the whole brain. In an early study (Kriegeskorte et al., 2007), searchlight was used to detect information that distinguishes between face tokens in the right anterior temporal lobe. The faces that were distinguished, though, were of different genders. A more recent study (Nestor et al., 2011) used searchlight and individuated information that distinguishes between face tokens of the same gender in the right anterior temporal lobe and posterior temporal cortex bilaterally.

**FIGURE 1:**
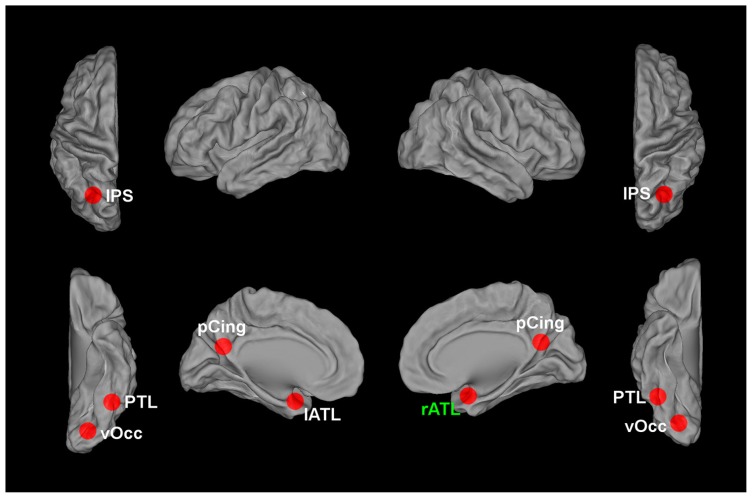
**Brain regions encoding information that contributes to the classification between different face tokens corresponding to different individuals**. vOcc, ventral occipital cortex; PTL, posterior temporal lobe; ATL, anterior temporal lobe; pCing, posterior cingulate; IPS, intraparietal sulcus. The current evidence indicates the right ATL, marked in green, as the most likely candidate region for encoding invariant representations of face identity.

Another method that can be used to individuate information that distinguishes between face tokens is recursive feature elimination (RFE), a type of MVPA (De Martino et al., 2008; Formisano et al., 2008). RFE has advantages (and some disadvantages) with respect to both ROI-based and searchlight methods. RFE can individuate information that is distributed beyond the extent of a searchlight sphere. It does not require that a set of contiguous voxels classify the different conditions significantly above chance; that is, informative voxels can be anywhere in the brain. This also means that feature selection approaches do not require making arbitrary choices about the size and shape of the regions within which to search for information. In addition, RFE requires that the individuated voxels contribute themselves to the discrimination, while in the case of searchlight an individuated voxel does not necessarily contribute to the discrimination: as long as other voxels within the sphere provide significant classification accuracy, the voxel will appear in the searchlight map, even if the voxel itself is not informative (this is especially true for SVM-based searchlight, see Etzel et al., 2013). The main disadvantage of RFE is that in its current form it allows localization of voxels that contribute to a given classification, but unlike searchlight and representational similarity analysis (RSA) it does not allow localization of regions based on a match between a neural dissimilarity matrix and a dissimilarity matrix hypothesized by the experimenter. However, for the purpose of localization of regions involved in the representation of face tokens this is not a major concern. To date, RFE has produced promising results for the localization of regions that distinguish between face tokens that depict different people (**Figure 1**), allowing localization of informative voxels for the discrimination between gender-matched faces in occipitotemporal and anterior temporal regions (Nestor et al., 2011; Anzellotti et al., 2013), and in the posterior cingulate and the posterior intraparietal sulcus (Anzellotti and Caramazza, 2014).

In sum, regions that distinguish between face tokens that depict different people have been found in occipitotemporal cortex bilaterally, in the anterior temporal lobes, in posterior cingulate and in bilateral IPS. Very recent studies (Cowen et al., 2014; Nestor et al., 2014) adopted principal component analysis (PCA) and independent component analysis (ICA) to investigate classification for larger numbers of face tokens, going beyond the small number of identities used in most studies to date.

## INVARIANT FACE REPRESENTATIONS

Regions that distinguish between face tokens that depict different people are candidate regions for representing face identity, but not all of them necessarily encode representations of face identity. To individuate regions that represent face identity, it is important to investigate whether they encode invariant face representations. Studies investigating the invariance of face representations typically look for evidence of commonalities among representations of different face tokens that depict the same person. For this reason, it is particularly important to carefully control the stimuli used because the presence of commonalities in the low-level properties of different face tokens depicting a same person can lead to illusory invariance effects. Equating the average luminance, color and texture in the whole image is often insufficient as a control because visually responsive neurons at several stages of processing have local receptive fields that do not encompass the entire image. These challenges can be overcome by generating stimuli with computer graphics. Using computer graphics permits the careful control of the low-level differences between face tokens at a local level (Anzellotti et al., 2013; Anzellotti and Caramazza, 2014). Since even cartoon faces elicit strong responses in face-selective neurons (Freiwald et al., 2009), it is unlikely that the use of realistic 3D renderings of faces would bias the results with respect to the use of photographs.

fMRI-adaptation can be used not only to individuate regions sensitive to differences in identity, but also to search for commonalities among representations of different face tokens that depict a same person. If a region encodes invariant face representations, the representations of different face tokens depicting the same person should overlap more than the representations of face tokens depicting different people, and therefore more fMR-A should be observed for the presentation of different face tokens that depict a same person than face tokens of different persons. One problem with the underlying assumptions motivating the use of fMR-A to study invariant face representations is that even if we accept that regions encoding invariant face representations should show fMR-A for the presentation of different face tokens depicting a same person, it does not follow that all regions that show fMR-A for the presentation of different face tokens depicting a same person encode invariant face representations. One way in which a region could show fMR-A for different face tokens depicting a same person despite encoding non-invariant face representations is through top-down influences. Via top-down influences, recognition of two different images as tokens depicting a same identity could lead to reduced activity not only in regions encoding invariant representations but also in early visual regions. Whether or not reduction in neural activity due to repetition can occur as a consequence of top-down influences is controversial (Xiang and Brown, 1998; Schendan and Kutas, 2003).

Several studies investigated invariant face representations using fMR-A, with mixed results: some studies found evidence for adaptation (Vuilleumier et al., 2002) while others did not (Pourtois et al., 2005). Ewbank and Andrews (2008) found fMR-A for repetition of face identity across different viewpoints in FFA when presenting familiar faces, but not when presenting novel faces. The likelihood of observing adaptation across different face tokens depicting a same person in fMR-A studies seems to be a function of the duration of the lag between two stimuli, with longer lags leading to more invariance in some studies (Andresen et al., 2009), but it remains unclear what are the mechanisms at the basis of this phenomenon. A recent study (Mur et al., 2010) found fMR-A for the repetition of face identity across different viewpoints in several regions, including early visual cortex. Given the current understanding of representations in early visual cortex, it is unlikely that this region carries invariant face representations. Findings such as this suggest that fMR-A can occur due to top-down influences.

To overcome the interpretative challenges that arise in fMR-A studies, invariant face representations have been investigated with MVPA. Experiments designed to investigate invariance with MVPA typically involve the presentation of multiple different tokens (e.g., different facial expressions, different viewpoints) of each face identity. The BOLD responses to those face tokens are then split into a subset used for the training of a classifier (for instance a support vector machine), and a subset used for the testing of the performance of the trained classifier. A possible approach is to split the data into subsets so that each part contains responses to all stimuli shown. In this case, the training and testing subsets contain the BOLD signal in response to *different* presentations of the *same identical* images. This analysis approach is *not* circular (data from different runs are used for the training and testing of classifiers), but since responses to the same images are used for training and testing, the classifier could potentially achieve significant classification accuracy relying on representations that are not invariant.

Despite these remarks, a recent study (Nestor et al., 2011) used this approach and found accuracies significantly above chance in FFA but at chance in early visual cortex for the classification of face identity in the presence of different facial expressions (Nestor et al., 2011). The robust classification accuracies obtained in this study (Nestor et al., 2011) are probably due to the contribution of invariant representations. However, other studies reported significant classification accuracy for faces seen from different viewpoints even in early visual cortex when using this method (Anzellotti et al., 2013). This is in contrast with the current understanding of representations in early visual cortex, and suggests that the conclusions obtained with this method should be interpreted with caution.

A more stringent method that overcomes the concerns discussed above consists in splitting the data into subsets so that the responses to different viewing conditions are included in the training and the testing set. In this case, the training and testing subsets contain the BOLD signal in response to *different* images. Using this method, classification across different viewpoints was at chance in early visual cortex, but was significant in other ventral stream regions (Anzellotti et al., 2013). In particular, even when using the responses to different stimuli for training and testing, and controlling carefully the “low-level” properties of images, significant classification generalizing across viewpoints was observed in both occipitotemporal and anterior temporal regions (Anzellotti et al., 2013). However, significant classification does not directly imply that a region carries representations of identity. The extent to which representations are invariant to transformations may vary, and a brain region could show invariance for some image transformations that humans can generalize across, but not for others. According to our definitions, such a representation would count as an invariant representation, but not as a representation of face identity.

Individuating significant classification accuracy across some specific transformations in multiple brain regions does not imply that the regions encode the same kind of representations. Therefore, occipitotemporal regions and anterior temporal regions might still encode different representations. To test this, a recent experiment investigated whether representations in different brain regions encoded information about face identity generalizing across different face halves (Anzellotti and Caramazza, 2014). For this manipulation, invariance was only found in the right anterior temporal lobe, and not in occipitotemporal cortex.

In the process of generating increasingly invariant representations, some information about identity-irrelevant differences between face tokens might be discarded or represented implicitly (DiCarlo and Cox, 2007). For this reason, the study of how and where identity-irrelevant information (e.g., information about viewpoint, illumination, and so on) is encoded can be seen as a complementary investigation to the study of invariance. Several studies provide evidence that identity-irrelevant information declines moving from posterior to anterior regions in the ventral stream (Kietzmann et al., 2012; Anzellotti and Caramazza, 2014; see Freiwald and Tsao, 2010 for similar evidence in monkeys, and Yovel and Freiwald, 2013 for a discussion of issues of homology). However, some identity-irrelevant information might still be present in more anterior regions (DiCarlo and Maunsell, 2003; Kravitz et al., 2008).

## CONCLUSION

Investigating the neural mechanisms underlying the recognition of face identity in humans is challenging, but the continuous development and improvement of design and analysis techniques has allowed the localization of representations that distinguish between face tokens depicting different people, and to begin to investigate their invariance. Given the current state of neuroimaging evidence, one region seems to encode face representations showing greatest invariance: the right anterior temporal lobe (Anzellotti et al., 2013; Anzellotti and Caramazza, 2014). This conclusion is consistent with neuropsychological evidence of deficits for face recognition after damage to the right anterior temporal lobe (Tranel et al., 1997), and with electrophysiology studies in monkeys (Freiwald and Tsao, 2010). However, it is important to note that current evidence does not establish that the right anterior temporal lobe is the only locus of face identity recognition: bilateral deficits are frequent in the anterior temporal lobes, and thus it remains possible that the left anterior temporal lobe also contributes, although to a lesser extent, to the recognition of face identity. In previous studies, the anterior temporal lobes have been implicated in semantic knowledge (Hodges et al., 1992; Tsukiura et al., 2002; Patterson et al., 2007). Invariant face representations could play an important role to link perceptual inputs to semantic knowledge about people.

Invariance does not appear only in the anterior temporal lobe, but builds up gradually, being present already to some extent in occipitotemporal regions (Kietzmann et al., 2012; Anzellotti et al., 2013; see Freiwald and Tsao, 2010 for consistent electrophysiology findings in monkeys), suggesting different roles for occipitotemporal and anterior temporal cortex for the recognition of face identity.

## Conflict of Interest Statement

The authors declare that the research was conducted in the absence of any commercial or financial relationships that could be construed as a potential conflict of interest.
